# Surgery in Mitral Stenosis

**Published:** 1902-04-05

**Authors:** 


### SURGERY IN MITRAL STENOSIS.

The progress of surgery of late years has been so rapid that no corner of the body seems exempt from operation. Sir Thomas Lauder Brunton has recently asked the surgical world whether it is impossible to relieve the hopeless misery and helplessness of advanced stenosis of the mitral valve by an incision, a tenotome being inserted through the auricle or ventricle so as to divide the stricture either parallel to the flaps of the valve or at right angles to them. He has experimented on the dead body, both in men and animals, and is now proceeding to perform the operation on chloroformed animals to ascertain its feasibility and dangers. In the human being the method of exposing the organ would be by incisions from the left side of the sternum along the lower borders of the third and fifth ribs for some three inches. A vertical incision would join these two, and the ribs would be folded back over the sternum, leaving the pericardium to be opened with due precaution. With sufficient skill and dexterity and in spite of the rhythmic expansion of the thorax and the pulsation of the heart itself it may be possible to introduce a tenotome through the heart wall and then to cut through the stricture in the valve. The bleeding must be completely stopped before the pericardium is closed up, for, as he points out, the heart's action is very easily embarrassed by blood or other fluid in that sac. In discussing this proposal one prominent surgeon at least has declared his belief in the practicability of the operation on these lines. The *Lancet*, while confessing how feeble *a priori* arguments have been when advanced against new operations on the uterus, brain, and spleen, thinks that the difficulties of such an operation are almost insuperable when we consider the rapidity of the manipulations required and the intricacy of the entrance between the deformed cusps of the valve. Then, too, if the edges of the orifice were incised, what is to prevent them from healing up and contracting more tightly than before, as in the urethra or the webs between fingers? There can be no subsequent passage of a bougie to prevent this mischance, and the additional cicatrix might only intensify the original mischief. Again, if the operation succeeded in remedying the stenosis by converting the lesion into a regurgitation, would the patient be any better off? To this latter objection indeed it may be replied that the results of advanced stenosis are far worse than those of regurgitation, and the destruction of the contracted ring would not in any ordinary case hinder muscular contraction from closing the valve. T. Fisher points out that the removal of the stricture would not necessarily relieve the block, for the heart wall in an advanced case is often greatly sclerosed and incapable of benefiting by it. Then, too, would there be any danger of wounding the coronary arteries or their branches? Should the operator aim at slightly nicking the edges of the stricture in various directions or incise it thoroughly on one side? A laryngeal surgeon, P. W. Williams, suggests the introduction of an instrument consisting of two or more blades which, when introduced within the stricture, could be separated to the required extent by a rack, so that the division could be made to any definite extent calculated beforehand. Indeed, the proposal is one which seems to abound in all sorts of possibilities and indeed of difficulties. Only prolonged work on the dead body and careful experiment by skilled surgeons can surmount the preliminary obstacles. At present it only amounts to a lofty aspiration well worthy of success.

